# Supplementary material for: Single-Cell DNA Sequencing Reveals an Evolutionary Pattern of CHIP in Transplant Eligible Multiple Myeloma Patients
Source: Cells. 2024 Apr 9;13(8):657. doi: 10.3390/cells13080657 (PMC11049155; doi:10.3390/cells13080657)
Supplement: Supplementary file 1 [file cells-13-00657-s001.zip › cells-2905095-supplementary.pdf]

Supplementary Figures

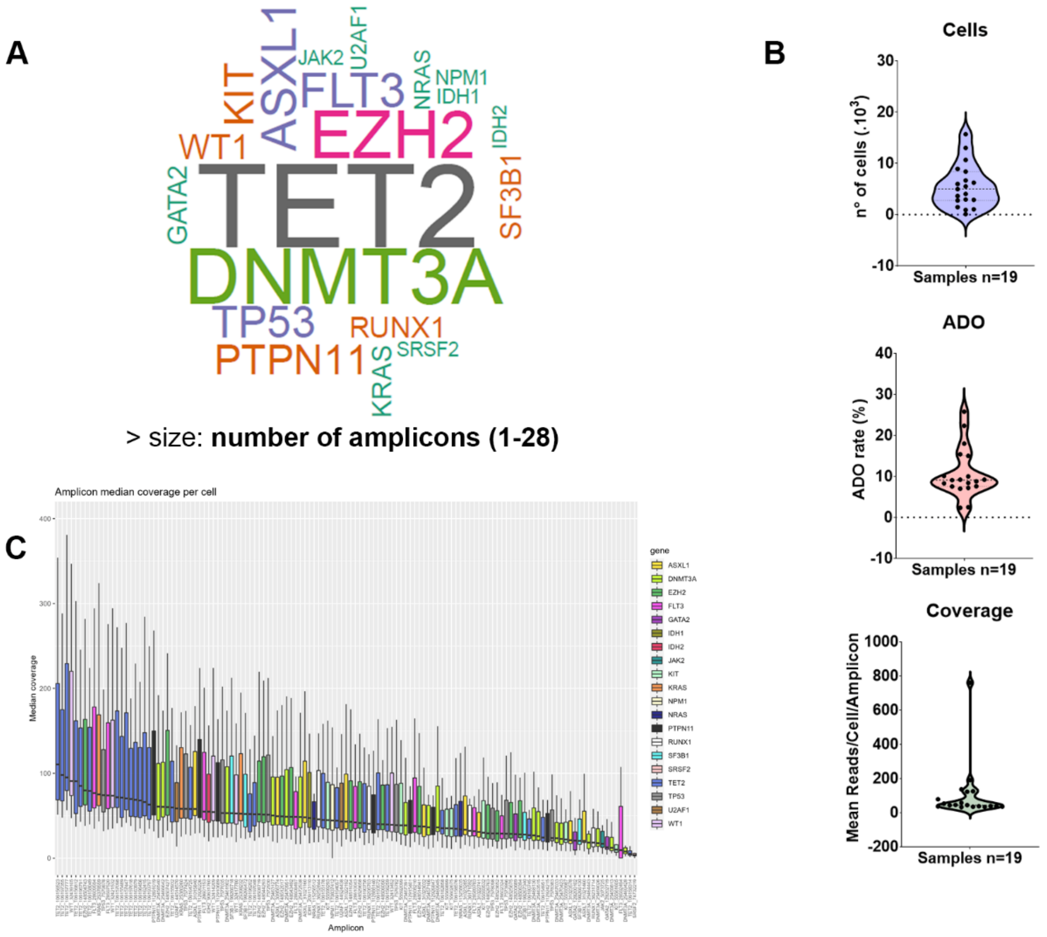

Supplementary Figure 1

Supplementary Figure S1. Single-cell DNA panel coverage and metrics of patient samples processed with the Tapestri platform for single-cell DNA sequencing. (**A**) The 20 genes included in the 127-amplicon panel, which covers reported mutational hotspots (SNVs and small indels) in acute myeloid leukemia. The text size is proportional to the number of amplicons per gene. Details of the genomic regions covered by the panel are in supplemental Table 1. (**B**) Total number of sequenced cells (top violin plot colored in violet), average allele dropout rate (ADO) per sample (middle violin plot colored in red), and average reads per amplicon per cell (bottom violin plot colored in green). Each point represents a sample (n=19) from 1 of the 12 MM patients. Median value is shown by a horizontal line. (**C**) Bar plot with the distribution of sequencing reads across the 127 amplicons. Each bar represents an amplicon, and the height of the bar shows the median number of raw sequenced reads per cell (n=19 samples). Amplicon name is shown for all the amplicons.

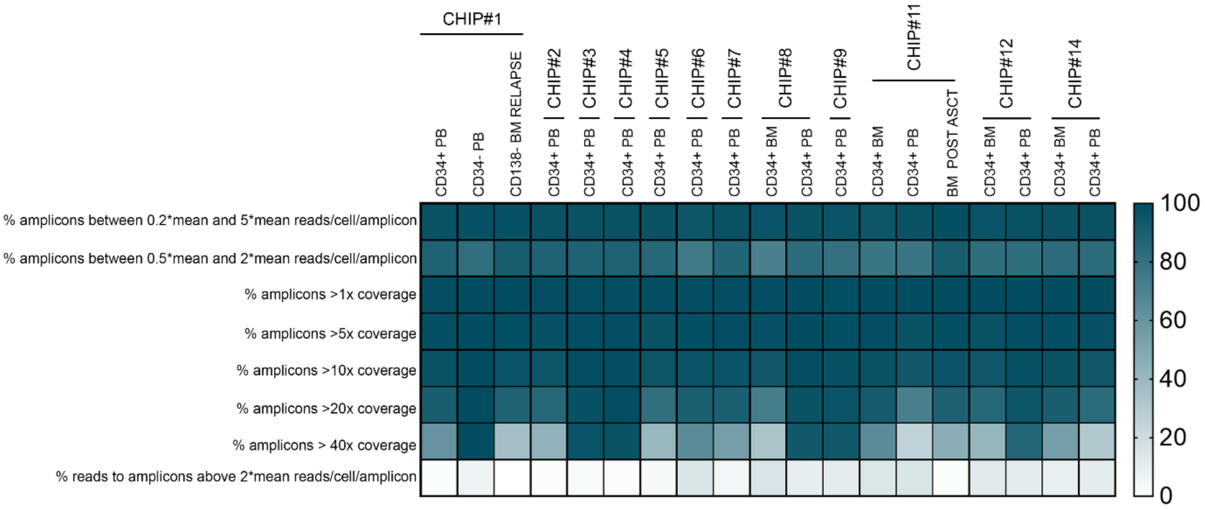

Supplementary Figure 2

Supplementary Figure S2. Panel performance for all patient's samples processed with the Tapestri platform for single-cell DNA sequencing. The AML panel used includes 127 amplicons and the heatmap shows the total coverage for all samples analysed (n=19). Each column represents a sample, and each row represents the relative percentage of reads/cell/amplicon for any given samples.

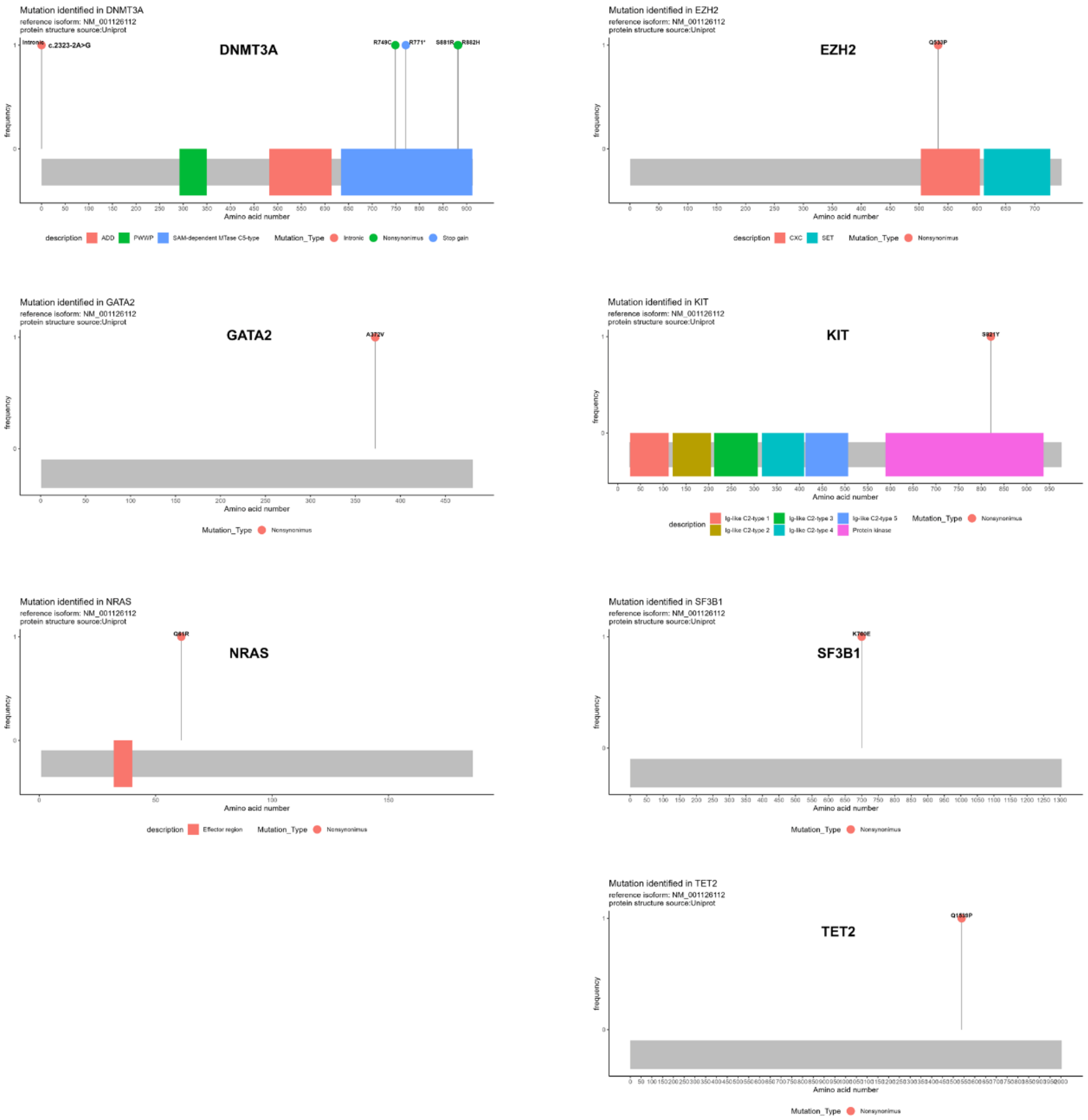

Supplementary Figure 3

Supplementary Figure S3. CHIP pathogenic mutations identified in MM patients in stem cells compartment at the time of transplant. Lolli plot showing the somatic mutation spectra throughout the protein sequence of DNMT3A, EZH2, GATA2, NRAS, KIT, SF3B1 and TET2. The scale bar represents the length (amino acids) of the protein sequence, and the corresponding domains are

indicated with coloured boxes. The corresponding variant name is shown on the top of the lolli plot specifying the coding impact.

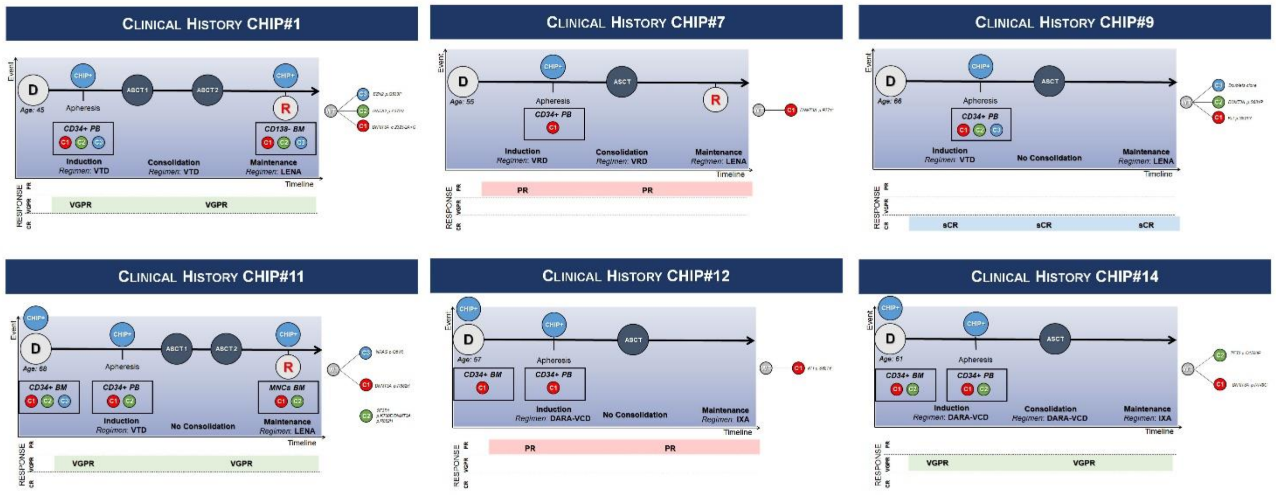

Supplementary Figure 4

Supplementary Figure S4. Clinical history and CHIP mutated clones for CHIP+ patients. D=Diagnosis, R=Relapse, ASCT=Autologous stem cell transplantation, CD34+BM=bone marrow stem cells, CD34+ PB=stem cells from apheresis product, CD138- BM=bone marrow CD138- cells fraction, MNCs BM=bone marrow mononuclear cells, VTD=bortezomib-thalidomide-dexamethasone, VCD=bortezomib-cyclophosphamide-dexamethasone, VRD=bortezomib-lenalidomi-dexamethasone, DARA=daratumumab, LENA=lenalidomide, IXA=ixazomib.

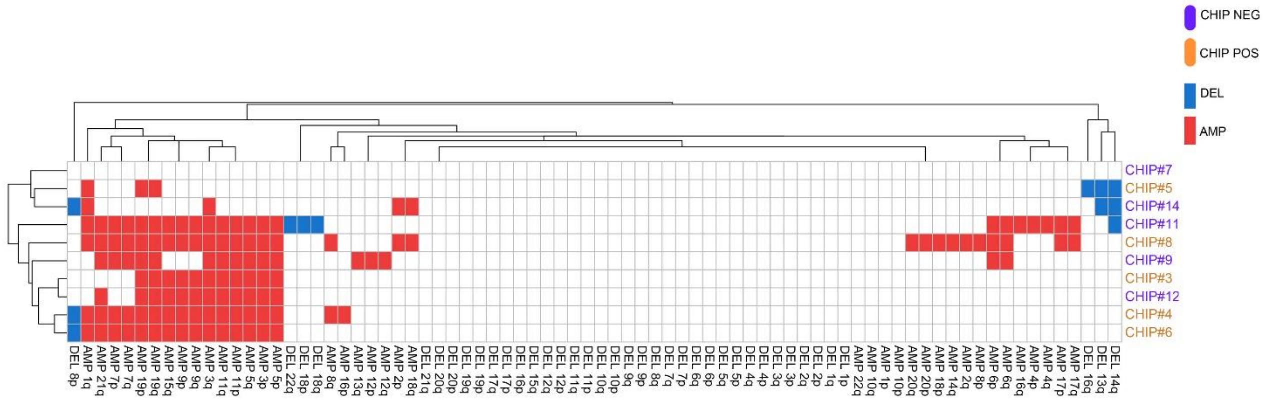

Supplementary Figure 5

Supplementary Figure S5. Copy Number Alterations (CNAs) of myeloma patients. On the CD138+ enriched fractions a genomic characterization by Ultra Low Pass-Whole Genome Sequencing (ULP-WGS) to evaluate whole genome's Copy Number Alterations (CNAs) profile were performed. ULP-WGS (0.1X coverage) was performed on genomic DNA samples from BM-PCs CD138+ cell fractions extracted by Maxwell® (Promega Italia Srl, Milan, Italy) using Maxwell® 16 LEV Blood DNA Kit. Library preparation was completed with SMARTer® ThruPLEX® DNA-Seq kit (Takara Bio, Mountain View, CA, USA). Sequencing was conducted on NextSeq 500 (Illumina Inc, San Diego, CA, USA), and IchorCNA was employed to assess CNAs profile. For CHIP#1 and CHIP#2, we were unable to assess the CNAs status due to insufficient material available for analysis.

Supplementary Table S1. Panel Targets

Gene	Exon	Protein-level Variants	cDNA Variants
ASXL1	3	G67	
	4	K85	
	11	R417; Q428; QA491; P511; A530	
	12	W583; Q588; R606; A611; K618; A772; L775; Q778; R786; T787; E797; A809; W898; G927; W960; K982; V1060	
DNMT3A	7	D268	
	8	P307; S312; W313; R320; W327	
	9	L344; L347; R366	
	10	W409; L422	
	11	K429; Y436; E444; A447	
	13	C494; C497; G511; M513	
	14	Q527; C540; G543; L547; G550; C554; C555	
	15	E561; W581; L595; R598; Q606; D614	
	16	R635; L637	
	17	L653; Y660; A662; S669; I681; D686; R688; V690	
	18	G699; D702; I705; G707; N711; L713; S714; V716; P718	
	19	F732; R736; R749; F751; F752; A760; S770; R771; L773; E774	
	20	P777; R792; W795; G796; N797; M801; R803	
	21	E817; H821	
	22	K829; T835; N838; Q842; P849; M852; E863	
	23	F868; D876; N879; M880; S881; R882; Q886; W893; I898; R899; P904	
	Intron		c.640-1437G>A; c.1429+2T>C; c.1667+1G>A; c.2408+5G>A; c.2479-1G>T
EZH2	2	M1; V13; Q28	
	3	N57	
	4	M121	
	5	H129; I131; Y133; Y153; G155	
	6	R207	
	8	E249; G266	
	10	T374	
	14	C548	
	15	K568; T573	
	16	G628; E645; Y646; C647; G648	
	17	A656; D664; N675	
	18	R684; R690; N693; H694; S695	
	19	Y731	
	20	D737; I744; E745	
FLT3	11	E444; S451	
	12	V491; Q494; A506; I507; S519; S531	
	13	I548	
	14	Y572; L576; V592; F594	
	16	M659; K663; M664; N676; A680	
	17	Q730	
	19	N781; T784	

	20	A814; V819; R834; D835; M837; S838; D839; N841; Y842	
	21	A856; T866	
GATA2	4	P304; N317; A318; G320; L321; R330	
	5	T358; L359; R361; R362; A372	
	6	R398	
IDH1	4	R132	
IDH2	4	R140; R172	
JAK2	14	V617	
KIT	2	P34; D52	
	5	D265	
	9	G487; G498	
	11	M552; W557; L576	
	12	T594; A617; M618; P627	
	17	D816; D829; N822; Y823	
	18	A837	
KRAS	2	G12; G13; H27	
	3	T58; Q61	
	4	K117; A146	
NPM1	11	L287; W290	c.860_861lin-sCTGC; c.861_862lin-sTGCA; c.863_864lin-sCATG; c.867_868lin-sAGGA;
NRAS	2	G12; G13	
	3	Q61	
PTPN11	3	N58; G60; D61; E69; F71; A72; T73; E76; Q79	
	4	E139	
	7	F285	
	8	M311	
	11	D437	
	12	A461; G464	
	13	P491; S502; G503; T507; Q510	
RUNX1	5	G127; E138; S141; A142; R145; R157; D160; R162; G165; R166; S167; G168R; K171	
	6	V197; D198; S200; R201; R204	
	7	R207; G217; S226; E229; R250; N260; M267	
	8	Y281; P298; E316	
	Intron		c.805+1G>A
SF3B1	14	G605; S611; R625; H662; K666; A672	
	15	K700; V701; I704	

	16	G742; A749; R775; E776; D781	
	17	D799	
	Intron		c.2224-1G>A
**SRSF2**	2	R167	
**TET2**	3	V9; E10; T27; L34; P46; Q80; G92; R96; D143; S145; S152; P174; E283; N312; Q325; Q414; Q147; Q481; R544; Q574; S588; Q591; Q622; Q635; H682; S714; Q734; Q810; S794; R814; S825; G898; Q913; Q916; P491; Q966; P989; W1003; Q1020; Q1053; Q1083; E1106; L1119	
	4	E1144; Y1148; N1156; R1167	
	5	Q1170; E1178; Q1191; C1193; W1198	
	6	S1203; S1204; R1214; R1216; D1242; Y1245; Y1255; R1261; R1262	
	7	C1271; F1287; G1288; C1289; Y1294; C1298; E1318	
	8	L1322; E1323; L1340	
	9	R1359; H1366; C1378; H1380	
	10	C1396; E1401; R1404; R1440; R1452; R1465; S1486; L1511; A1512	
	11	R1516; Q1532; Q1539; Q1542; Q1548; Q1624; Q1652; V1718; L1721; P1723S; R1739; L1740; H1757; I1762; C1811; L1819; Q1828; G1861; S1898; V1900; H1904; H1912; A1919; R1926; P1941; P1962; R1966; P1988; Y1998; R2000; I2002	
	Intron		c.3409+70G>A
**TP53**	2		c.-87G>C; c.-93A>G
	5	L91; K93; F95; T101; V104; W107; V108; G115; R119; Y124; K125; Q126; H129; V134; R136; C137	
	6	H154; I156; R157; V164; Y166; R174; S176; Y181; P184	
	7	Y195; VM198; C199; N200; S202; C203; G206; R209; I215; E219	
	8	G223; L226; G227; R228; V233; R234; C236; A237; P239; D242; R244; R251	
	10	F302; R303	
	11	F346	
	Intron		c.259-2A>G; c.259-1G>A; c.443-1G>A; c.876+1G>A; c.877-1G>A;
**U2AF1**	2	R28; S34; R35	
	6	R156; Q157	
	7	R188	

WT1	4	L299; M302; K321	
	6	G356	
	7	R374; R375; S386	
	8	R435; R436; R439; Q442	
	9	R463; R467; D469; H470	
	Intron		c.1115_1264+1del; c.1264+3_1264+4insT

Supplementary Table S2. Called somatic Variants. All somatic coding variants identified in all samples analyzed (n=19). Overall 235 nucleotide variants have been called. SNV=Single Nucleotide Variant.

Sample	Sample Type	Gene	Chromosome	Variant	Function	Coding Impact	Variant Type	cDNA	Protein Change	Genotyped Cells	Mutated Cells	VAF by cell count (%)	VAF by read count (%)
**CHIP14**	CD34+ BM	ASXL1	20	ASXL1:chr20:31015949:T/G	Coding	Missense	SNV	c.271T>G	p.S91A	4081 (54%)	101 (2%)	1.2	8.7
**CHIP12**	CD34+ PB	ASXL1	20	ASXL1:chr20:31015949:T/G	Coding	Missense	SNV	c.271T>G	p.S91A	3066 (56%)	55 (2%)	0.9	9.1
**CHIP9**	CD34+ PB	ASXL1	20	ASXL1:chr20:31021202:A/C	Coding	Missense	SNV	c.1201A>C	p.T401P	2160 (78%)	37 (2%)	0.9	5.1
**CHIP9**	CD34+ PB	ASXL1	20	ASXL1:chr20:31021207:A/C	Coding	Synonymous	SNV	c.1206A>C	p.R402=	2032 (74%)	42 (2%)	1.0	5.2
**CHIP8**	CD34+ PB	ASXL1	20	ASXL1:chr20:31021461:C/T	Coding	Missense	SNV	c.1460C>T	p.A487V	1495 (52%)	703 (47%)	23.6	17.0
**CHIP9**	CD34+ PB	ASXL1	20	ASXL1:chr20:31021479:A/C	Coding	Missense	SNV	c.1478A>C	p.E493A	1705 (62%)	31 (2%)	0.9	5.4
**CHIP4**	CD34+ PB	ASXL1	20	ASXL1:chr20:31021634:C/T	Coding	Missense	SNV	c.1633C>T	p.R545C	778 (86%)	11 (1%)	0.7	1.7
**CHIP3**	CD34+ PB	ASXL1	20	ASXL1:chr20:31021634:C/T	Coding	Missense	SNV	c.1633C>T	p.R545C	1216 (84%)	16 (1%)	0.7	1.6
**CHIP5**	CD34+ PB	ASXL1	20	ASXL1:chr20:31022396:G/A	Coding	Synonymous	SNV	c.1881G>A	p.A627=	3013 (50%)	80 (3%)	1.3	2.1
**CHIP8**	CD34+ BM	ASXL1	20	ASXL1:chr20:31022396:G/A	Coding	Synonymous	SNV	c.1881G>A	p.A627=	6999 (55%)	160 (2%)	1.1	2.0
**CHIP4**	CD34+ PB	ASXL1	20	ASXL1:chr20:31022938:C/A	Coding	Missense	SNV	c.2423C>A	p.P808H	862 (95%)	818 (95%)	50.2	51.2
**CHIP9**	CD34+ PB	ASXL1	20	ASXL1:chr20:31023033:C/G	Coding	Missense	SNV	c.2518C>G	p.P840A	1909 (69%)	39 (2%)	1.0	4.2
**CHIP14**	CD34+ BM	ASXL1	20	ASXL1:chr20:31023033:C/G	Coding	Missense	SNV	c.2518C>G	p.P840A	4963 (66%)	117 (2%)	1.2	1.8
**CHIP12**	CD34+ PB	ASXL1	20	ASXL1:chr20:31023033:C/G	Coding	Missense	SNV	c.2518C>G	p.P840A	3920 (72%)	115 (3%)	1.5	2.2
**CHIP11**	CD34+ PB	ASXL1	20	ASXL1:chr20:31023033:C/G	Coding	Missense	SNV	c.2518C>G	p.P840A	5744 (61%)	160 (3%)	1.4	1.9
**CHIP11**	CD34+ BM	ASXL1	20	ASXL1:chr20:31023033:C/G	Coding	Missense	SNV	c.2518C>G	p.P840A	798 (78%)	24 (3%)	1.5	2.5
**CHIP9**	CD34+ PB	ASXL1	20	ASXL1:chr20:31023045:A/C	Coding	Missense	SNV	c.2530A>C	p.T844P	1434 (52%)	55 (4%)	1.9	6.6
**CHIP8**	CD34+ PB	ASXL1	20	ASXL1:chr20:31023122:T/G	Coding	Synonymous	SNV	c.2607T>G	p.G869=	1505 (52%)	29 (2%)	1.0	9.0
**CHIP9**	CD34+ PB	ASXL1	20	ASXL1:chr20:31023126:T/G	Coding	Missense	SNV	c.2611T>G	p.S871A	1653 (60%)	42 (3%)	1.3	5.8
**CHIP1**	CD34+ PB	ASXL1	20	ASXL1:chr20:31023286:C/T	Coding	Missense	SNV	c.2771C>T	p.P924L	3160 (87%)	38 (1%)	0.6	1.1
**CHIP9**	CD34+ PB	ASXL1	20	ASXL1:chr20:31023362:T/G	Coding	Synonymous	SNV	c.2847T>G	p.G949=	2031 (74%)	141 (7%)	3.5	7.5
**CHIP9**	CD34+ PB	ASXL1	20	ASXL1:chr20:31023714:T/G	Coding	Missense	SNV	c.3199T>G	p.S1067A	1417 (51%)	35 (2%)	1.2	7.3
**CHIP9**	CD34+ PB	DNMT3A	2	DNMT3A:chr2:25457213:A/C	Coding	Missense	SNV	c.2674T>G	p.S892A	1607 (58%)	87 (5%)	2.7	6.6
**CHIP9**	CD34+ PB	DNMT3A	2	DNMT3A:chr2:25457221:A/C	Coding	Missense	SNV	c.2666T>G	p.L889R	1897 (69%)	45 (2%)	1.2	5.9

**CHIP11**	BM post ASCT	DNMT3A	2	DNMT3A:chr2:25457242:C/T	Coding	Missense	SNV	c.2645G>A	p.R882H	5376 (88%)	3985 (74%)	39.6	38.5
**CHIP11**	CD34+ PB	DNMT3A	2	DNMT3A:chr2:25457242:C/T	Coding	Missense	SNV	c.2645G>A	p.R882H	6843 (72%)	5312 (78%)	41.3	37.1
**CHIP11**	CD34+ BM	DNMT3A	2	DNMT3A:chr2:25457242:C/T	Coding	Missense	SNV	c.2645G>A	p.R882H	837 (81%)	521 (62%)	35.2	34.5
**CHIP9**	CD34+ PB	DNMT3A	2	DNMT3A:chr2:25457246:T/G	Coding	Missense	SNV	c.2641A>C	p.S881R	1661 (60%)	320 (19%)	9.6	13.4
**CHIP1**	CD34- PB	DNMT3A	2	DNMT3A:chr2:25457246:T/G	Coding	Missense	SNV	c.2641A>C	p.S881R	70 (71%)	20 (29%)	14.3	16.8
**CHIP8**	CD34+ PB	DNMT3A	2	DNMT3A:chr2:25457246:T/G	Coding	Missense	SNV	c.2641A>C	p.S881R	2555 (88%)	50 (2%)	1.0	4.3
**CHIP1**	CD34- PB	DNMT3A	2	DNMT3A:chr2:25457269:TG/T	NMD, coding(2)	Frameshift	Deletion	c.2617del	p.H873Tfs*8	97 (99%)	2 (2%)	1.0	3.2
**CHIP12**	CD34+ PB	DNMT3A	2	DNMT3A:chr2:25462007:A/C	Coding, splicing	Synonymous	SNV	c.2400T>G	p.G800=	3241 (60%)	58 (2%)	0.9	6.6
**CHIP1**	CD34- PB	DNMT3A	2	DNMT3A:chr2:25462086:T/G	Intronic, splicing, splicing-ACMG(1)		SNV	c.2323-2A>C		61 (62%)	5 (8%)	4.1	4.6
**CHIP7**	CD34+ PB	DNMT3A	2	DNMT3A:chr2:25463182:G/A	NMD, coding(2)	Nonsense	SNV	c.2311C>T	p.R771*	4758 (96%)	134 (3%)	1.5	1.9
**CHIP14**	CD34+ PB	DNMT3A	2	DNMT3A:chr2:25463248:G/A	Coding	Missense	SNV	c.2245C>T	p.R749C	4973 (80%)	121 (2%)	1.2	3.3
**CHIP14**	CD34+ BM	DNMT3A	2	DNMT3A:chr2:25463248:G/A	Coding	Missense	SNV	c.2245C>T	p.R749C	6361 (85%)	100 (2%)	0.8	3.0
**CHIP1**	CD34- PB	DNMT3A	2	DNMT3A:chr2:25463595:T/G	Coding, splicing	Missense	SNV	c.2087A>C	p.Q696P	66 (67%)	9 (14%)	6.8	8.4
**CHIP1**	CD34- PB	DNMT3A	2	DNMT3A:chr2:25464452:G/A	Coding	Synonymous	SNV	c.2061C>T	p.V687=	93 (95%)	1 (1%)	0.5	3.5
**CHIP8**	CD34+ PB	DNMT3A	2	DNMT3A:chr2:25467131:T/G	Coding	Missense	SNV	c.1744A>C	p.N582H	1537 (53%)	41 (3%)	1.3	4.9
**CHIP1**	CD138- BM	DNMT3A	2	DNMT3A:chr2:25467453:A/C	Coding	Missense	SNV	c.1623T>G	p.C541W	4601 (54%)	131 (3%)	1.4	5.3
**CHIP9**	CD34+ PB	DNMT3A	2	DNMT3A:chr2:25467455:A/C	Coding	Missense	SNV	c.1621T>G	p.C541G	1660 (60%)	28 (2%)	0.8	3.5
**CHIP8**	CD34+ PB	DNMT3A	2	DNMT3A:chr2:25467487:T/G	Coding	Missense	SNV	c.1589A>C	p.D530A	1452 (50%)	30 (2%)	1.0	7.5
**CHIP4**	CD34+ PB	DNMT3A	2	DNMT3A:chr2:25469502:C/T	Coding	Synonymous	SNV	c.1266G>A	p.L422=	776 (86%)	694 (89%)	52.4	52.8
**CHIP3**	CD34+ PB	DNMT3A	2	DNMT3A:chr2:25469502:C/T	Coding	Synonymous	SNV	c.1266G>A	p.L422=	1269 (88%)	1157 (91%)	50.9	51.3
**CHIP1**	CD34+ PB	DNMT3A	2	DNMT3A:chr2:25469502:C/T	Coding	Synonymous	SNV	c.1266G>A	p.L422=	2103 (58%)	1985 (94%)	54.4	53.7
**CHIP1**	CD34- PB	DNMT3A	2	DNMT3A:chr2:25469502:C/T	Coding	Synonymous	SNV	c.1266G>A	p.L422=	81 (83%)	69 (85%)	46.9	43.7
**CHIP8**	CD34+ PB	DNMT3A	2	DNMT3A:chr2:25469502:C/T	Coding	Synonymous	SNV	c.1266G>A	p.L422=	1703 (59%)	1514 (89%)	52.2	51.2
**CHIP5**	CD34+ PB	EZH2	7	EZH2:chr7:148508780:G/C	Coding	Synonymous	SNV	c.1884C>G	p.G628=	5776 (97%)	5705 (99%)	50.2	51.1
**CHIP1**	CD138- BM	EZH2	7	EZH2:chr7:148511139:T/G	Coding	Missense	SNV	c.1763A>C	p.D588A	4782 (56%)	131 (3%)	1.4	6.5
**CHIP9**	CD34+ PB	EZH2	7	EZH2:chr7:148511145:T/G	Coding	Missense	SNV	c.1757A>C	p.D586A	1960 (71%)	36 (2%)	0.9	7.3
**CHIP6**	CD34+ PB	EZH2	7	EZH2:chr7:148511171:C/T	Coding	Synonymous	SNV	c.1731G>A	p.P577=	2558 (87%)	2463 (96%)	50.1	52.1

**CHIP11**	BM post ASCT	EZH2	7	EZH2:chr7:148512080:T/G	Coding	Missense	SNV	c.1598A>C	p.Q533P	3386 (55%)	124 (4%)	1.8	5.1
**CHIP1**	CD138- BM	EZH2	7	EZH2:chr7:148512080:T/G	Coding	Missense	SNV	c.1598A>C	p.Q533P	5084 (60%)	182 (4%)	1.8	4.8
**CHIP14**	CD34+ BM	EZH2	7	EZH2:chr7:148512080:T/G	Coding	Missense	SNV	c.1598A>C	p.Q533P	4309 (57%)	134 (3%)	1.6	4.8
**CHIP8**	CD34+ BM	EZH2	7	EZH2:chr7:148512080:T/G	Coding	Missense	SNV	c.1598A>C	p.Q533P	6765 (53%)	130 (2%)	1.0	3.2
**CHIP12**	CD34+ BM	EZH2	7	EZH2:chr7:148512080:T/G	Coding	Missense	SNV	c.1598A>C	p.Q533P	2175 (54%)	49 (2%)	1.1	3.5
**CHIP1**	CD34- PB	EZH2	7	EZH2:chr7:148514997:CTCT/C	Coding	In frame	Deletion	c.1209_1211del	p.E404del	72 (73%)	1 (1%)	0.7	4.8
**CHIP1**	CD34- PB	EZH2	7	EZH2:chr7:148515091:G/A	Coding	Missense	SNV	c.1118C>T	p.P373L	98 (100%)	1 (1%)	0.5	4.5
**CHIP1**	CD34- PB	EZH2	7	EZH2:chr7:148525854:T/C	Coding	Synonymous	SNV	c.603A>G	p.K201=	98 (100%)	2 (2%)	1.0	1.8
**CHIP1**	CD34- PB	EZH2	7	EZH2:chr7:148526901:C/T	Coding	Missense	SNV	c.403G>A	p.G135R	98 (100%)	1 (1%)	0.5	5.6
**CHIP6**	CD34+ PB	EZH2	7	EZH2:chr7:148526923:A/G	Coding	Synonymous	SNV	c.381T>C	p.V127=	1799 (61%)	1746 (97%)	48.5	32.3
**CHIP7**	CD34+ PB	EZH2	7	EZH2:chr7:148526923:A/G	Coding	Synonymous	SNV	c.381T>C	p.V127=	2887 (58%)	2726 (94%)	47.2	29.8
**CHIP4**	CD34+ PB	EZH2	7	EZH2:chr7:148526923:A/G	Coding	Synonymous	SNV	c.381T>C	p.V127=	588 (65%)	558 (95%)	47.4	28.9
**CHIP3**	CD34+ PB	EZH2	7	EZH2:chr7:148526923:A/G	Coding	Synonymous	SNV	c.381T>C	p.V127=	1101 (76%)	1075 (98%)	48.8	30.2
**CHIP11**	BM post ASCT	EZH2	7	EZH2:chr7:148526923:A/G	Coding	Synonymous	SNV	c.381T>C	p.V127=	3477 (57%)	3236 (93%)	46.5	28.7
**CHIP1**	CD34+ PB	EZH2	7	EZH2:chr7:148526923:A/G	Coding	Synonymous	SNV	c.381T>C	p.V127=	2291 (63%)	2189 (96%)	47.8	29.2
**CHIP14**	CD34+ BM	EZH2	7	EZH2:chr7:148526923:A/G	Coding	Synonymous	SNV	c.381T>C	p.V127=	4515 (60%)	4150 (92%)	46.0	29.4
**CHIP8**	CD34+ PB	EZH2	7	EZH2:chr7:148526923:A/G	Coding	Synonymous	SNV	c.381T>C	p.V127=	2079 (72%)	1960 (94%)	47.1	31.4
**CHIP12**	CD34+ PB	EZH2	7	EZH2:chr7:148526923:A/G	Coding	Synonymous	SNV	c.381T>C	p.V127=	3817 (70%)	3704 (97%)	48.5	33.1
**CHIP12**	CD34+ BM	EZH2	7	EZH2:chr7:148526923:A/G	Coding	Synonymous	SNV	c.381T>C	p.V127=	2148 (53%)	1994 (93%)	46.4	30.5
**CHIP11**	CD34+ PB	EZH2	7	EZH2:chr7:148526923:A/G	Coding	Synonymous	SNV	c.381T>C	p.V127=	5113 (54%)	4903 (96%)	47.9	31.8
**CHIP11**	CD34+ BM	EZH2	7	EZH2:chr7:148526923:A/G	Coding	Synonymous	SNV	c.381T>C	p.V127=	691 (67%)	664 (96%)	48.0	33.8
**CHIP9**	CD34+ PB	EZH2	7	EZH2:chr7:148529751:C/A	Coding	Missense	SNV	c.338G>T	p.W113L	1650 (60%)	35 (2%)	1.1	3.6
**CHIP9**	CD34+ PB	EZH2	7	EZH2:chr7:148529754:G/T	Coding	Missense	SNV	c.335C>A	p.S112Y	1586 (57%)	29 (2%)	0.9	4.1
**CHIP1**	CD34- PB	FLT3	13	FLT3:chr13:28601250:TG/T	NMD, coding(2)	Frameshift	Deletion	c.2181del	p.T728Lfs*53	93 (95%)	2 (2%)	1.1	7.5
**CHIP1**	CD34- PB	FLT3	13	FLT3:chr13:28608288:A/G	Coding	Missense	SNV	c.1768T>C	p.F590L	98 (100%)	1 (1%)	0.5	5.6
**CHIP1**	CD34- PB	FLT3	13	FLT3:chr13:28609667:C/T	Coding	Missense	SNV	c.1562G>A	p.G521D	98 (100%)	1 (1%)	0.5	6.1

**CHIP6**	CD34+ PB	GATA2	3	GATA2:chr3:128200690:G/A	Coding	Missense	SNV	c.1115C>T	p.A372V	2423 (82%)	30 (1%)	0.6	1.3
**CHIP7**	CD34+ PB	GATA2	3	GATA2:chr3:128200690:G/A	Coding	Missense	SNV	c.1115C>T	p.A372V	3454 (70%)	67 (2%)	1.0	1.5
**CHIP4**	CD34+ PB	GATA2	3	GATA2:chr3:128200690:G/A	Coding	Missense	SNV	c.1115C>T	p.A372V	768 (85%)	10 (1%)	0.7	1.7
**CHIP11**	BM post ASCT	GATA2	3	GATA2:chr3:128200690:G/A	Coding	Missense	SNV	c.1115C>T	p.A372V	4017 (66%)	105 (3%)	1.3	2.4
**CHIP1**	CD34+ PB	GATA2	3	GATA2:chr3:128200690:G/A	Coding	Missense	SNV	c.1115C>T	p.A372V	2549 (70%)	51 (2%)	2.4	1.6
**CHIP1**	CD138- BM	GATA2	3	GATA2:chr3:128200690:G/A	Coding	Missense	SNV	c.1115C>T	p.A372V	5010 (59%)	127 (3%)	1.3	2.6
**CHIP11**	CD34+ BM	GATA2	3	GATA2:chr3:128200690:G/A	Coding	Missense	SNV	c.1115C>T	p.A372V	674 (66%)	13 (2%)	1.0	1.6
**CHIP9**	CD34+ PB	GATA2	3	GATA2:chr3:128200787:A/T	Coding, splicing, splicing-ACMG(1)	Missense	SNV	c.1018T>A	p.S340T	1967 (71%)	181 (9%)	4.6	4.7
**CHIP1**	CD34- PB	IDH1	2	IDH1:chr2:209113159:AT/A	NMD, coding(2)	Frameshift	Deletion (homopolymer)	c.347del	p.N116Ifs*6	98 (100%)	1 (1%)	0.5	5.9
**CHIP4**	CD34+ PB	IDH1	2	IDH1:chr2:209113192:G/A	Coding	Synonymous	SNV	c.315C>T	p.G105=	864 (96%)	831 (96%)	49.7	50.4
**CHIP9**	CD34+ PB	IDH2	15	IDH2:chr15:90631828:A/C	Coding, splicing	Missense	SNV	c.525T>G	p.H175Q	1485 (54%)	49 (3%)	1.6	4.3
**CHIP1**	CD34- PB	JAK2	9	JAK2:chr9:5073752:T/A	Coding	Missense	SNV	c.1831T>A	p.L611I	96 (98%)	1 (1%)	0.5	6.1
**CHIP1**	CD34- PB	KIT	4	KIT:chr4:55561718:ACCAT/A	NMD, coding(2)	Frameshift	Deletion	c.120_123del	p.H40Qfs*6	93 (95%)	1 (1%)	0.5	3.4
**CHIP6**	CD34+ PB	KIT	4	KIT:chr4:55569900:A/T	Coding	Missense	SNV	c.767A>T	p.Q256L	2300 (78%)	50 (2%)	1.1	1.9
**CHIP5**	CD34+ PB	KIT	4	KIT:chr4:55569900:A/T	Coding	Missense	SNV	c.767A>T	p.Q256L	3316 (56%)	117 (4%)	1.8	2.1
**CHIP2**	CD34+ PB	KIT	4	KIT:chr4:55569900:A/T	Coding	Missense	SNV	c.767A>T	p.Q256L	6544 (54%)	255 (4%)	1.9	2.1
**CHIP14**	CD34+ BM	KIT	4	KIT:chr4:55569900:A/T	Coding	Missense	SNV	c.767A>T	p.Q256L	4988 (66%)	131 (3%)	1.3	1.8
**CHIP8**	CD34+ PB	KIT	4	KIT:chr4:55569900:A/T	Coding	Missense	SNV	c.767A>T	p.Q256L	2472 (86%)	44 (2%)	0.9	1.9
**CHIP12**	CD34+ PB	KIT	4	KIT:chr4:55569900:A/T	Coding	Missense	SNV	c.767A>T	p.Q256L	3880 (71%)	151 (4%)	1.9	2.1
**CHIP12**	CD34+ BM	KIT	4	KIT:chr4:55569900:A/T	Coding	Missense	SNV	c.767A>T	p.Q256L	2370 (59%)	42 (2%)	0.9	1.3
**CHIP11**	CD34+ PB	KIT	4	KIT:chr4:55569900:A/T	Coding	Missense	SNV	c.767A>T	p.Q256L	5025 (53%)	151 (3%)	1.5	1.5
**CHIP11**	CD34+ BM	KIT	4	KIT:chr4:55569900:A/T	Coding	Missense	SNV	c.767A>T	p.Q256L	709 (69%)	20 (3%)	1.4	2.1
**CHIP9**	CD34+ PB	KIT	4	KIT:chr4:55599336:C/A	Coding	Missense	SNV	c.2462C>A	p.S821Y	1463 (53%)	926 (63%)	31.6	23.8
**CHIP3**	CD34+ PB	KIT	4	KIT:chr4:55599336:C/A	Coding	Missense	SNV	c.2462C>A	p.S821Y	981 (68%)	27 (3%)	1.4	7.4
**CHIP2**	CD34+ PB	KIT	4	KIT:chr4:55599336:C/A	Coding	Missense	SNV	c.2462C>A	p.S821Y	10534 (87%)	183 (2%)	0.9	3.4
**CHIP11**	BM post ASCT	KIT	4	KIT:chr4:55599336:C/A	Coding	Missense	SNV	c.2462C>A	p.S821Y	4887 (80%)	133 (3%)	1.4	4.8
**CHIP1**	CD138- BM	KIT	4	KIT:chr4:55599336:C/A	Coding	Missense	SNV	c.2462C>A	p.S821Y	6809 (80%)	142 (2%)	1.0	3.8

**CHIP1**	CD34- PB	KIT	4	KIT:chr4:55599336:C/A	Coding	Missense	SNV	c.2462C>A	p.S821Y	54 (55%)	50 (93%)	46.3	26.7
**CHIP14**	CD34+ PB	KIT	4	KIT:chr4:55599336:C/A	Coding	Missense	SNV	c.2462C>A	p.S821Y	3937 (63%)	203 (5%)	2.6	6.1
**CHIP14**	CD34+ BM	KIT	4	KIT:chr4:55599336:C/A	Coding	Missense	SNV	c.2462C>A	p.S821Y	5479 (73%)	462 (8%)	4.2	8.8
**CHIP8**	CD34+ BM	KIT	4	KIT:chr4:55599336:C/A	Coding	Missense	SNV	c.2462C>A	p.S821Y	8445 (66%)	226 (3%)	1.3	3.9
**CHIP12**	CD34+ PB	KIT	4	KIT:chr4:55599336:C/A	Coding	Missense	SNV	c.2462C>A	p.S821Y	3424 (63%)	533 (16%)	7.8	14.4
**CHIP12**	CD34+ BM	KIT	4	KIT:chr4:55599336:C/A	Coding	Missense	SNV	c.2462C>A	p.S821Y	2500 (62%)	185 (7%)	3.7	7.4
**CHIP11**	CD34+ PB	KIT	4	KIT:chr4:55599336:C/A	Coding	Missense	SNV	c.2462C>A	p.S821Y	7001 (74%)	125 (2%)	0.9	2.9
**CHIP11**	CD34+ BM	KIT	4	KIT:chr4:55599336:C/A	Coding	Missense	SNV	c.2462C>A	p.S821Y	674 (66%)	60 (9%)	4.5	9.2
**CHIP6**	CD34+ PB	KIT	4	KIT:chr4:55602765:G/C	Coding	Synonymous	SNV	c.2586G>C	p.L862=	2591 (88%)	2505 (97%)	49.9	51.3
**CHIP2**	CD34+ PB	KIT	4	KIT:chr4:55602765:G/C	Coding	Synonymous	SNV	c.2586G>C	p.L862=	11162 (92%)	11061 (99%)	50.1	50.5
**CHIP8**	CD34+ PB	KIT	4	KIT:chr4:55602765:G/C	Coding	Synonymous	SNV	c.2586G>C	p.L862=	2550 (88%)	2436 (96%)	49.8	51.3
**CHIP8**	CD34+ BM	KIT	4	KIT:chr4:55602765:G/C	Coding	Synonymous	SNV	c.2586G>C	p.L862=	8154 (64%)	8092 (99%)	49.9	50.2
**CHIP8**	CD34+ PB	KRAS	12	KRAS:chr12:25380328:C/A	Coding	Missense	SNV	c.130G>T	p.V44L	1678 (58%)	110 (7%)	3.3	8.5
**CHIP12**	CD34+ PB	KRAS	12	KRAS:chr12:25380328:C/A	Coding	Missense	SNV	c.130G>T	p.V44L	4131 (76%)	122 (3%)	1.5	6.2
**CHIP6**	CD34+ PB	NRAS	1	NRAS:chr1:115256513:G/A	Coding	Synonymous	SNV	c.198C>T	p.A66=	2594 (88%)	40 (2%)	0.8	2.2
**CHIP7**	CD34+ PB	NRAS	1	NRAS:chr1:115256513:G/A	Coding	Synonymous	SNV	c.198C>T	p.A66=	4241 (86%)	72 (2%)	0.8	1.9
**CHIP4**	CD34+ PB	NRAS	1	NRAS:chr1:115256513:G/A	Coding	Synonymous	SNV	c.198C>T	p.A66=	765 (85%)	11 (1%)	0.7	3.1
**CHIP3**	CD34+ PB	NRAS	1	NRAS:chr1:115256513:G/A	Coding	Synonymous	SNV	c.198C>T	p.A66=	1150 (79%)	23 (2%)	1.0	3.3
**CHIP11**	BM post ASCT	NRAS	1	NRAS:chr1:115256513:G/A	Coding	Synonymous	SNV	c.198C>T	p.A66=	4666 (76%)	67 (1%)	0.7	2.8
**CHIP1**	CD34+ PB	NRAS	1	NRAS:chr1:115256513:G/A	Coding	Synonymous	SNV	c.198C>T	p.A66=	2981 (82%)	72 (2%)	1.2	2.3
**CHIP11**	CD34+ BM	NRAS	1	NRAS:chr1:115256513:G/A	Coding	Synonymous	SNV	c.198C>T	p.A66=	860 (84%)	11 (1%)	0.6	2.4
**CHIP11**	CD34+ BM	NRAS	1	NRAS:chr1:115256529:T/C	Coding	Missense	SNV	c.182A>G	p.Q61R	945 (92%)	19 (2%)	1.1	2.3
**CHIP11**	BM post ASCT	PTPN11	12	PTPN11:chr12:112891084:A/C	Coding	Missense	SNV	c.418A>C	p.S140R	5006 (82%)	166 (3%)	1.7	6.2
**CHIP1**	CD138- BM	PTPN11	12	PTPN11:chr12:112891084:A/C	Coding	Missense	SNV	c.418A>C	p.S140R	7432 (87%)	91 (1%)	0.6	5.0
**CHIP14**	CD34+ PB	PTPN11	12	PTPN11:chr12:112891084:A/C	Coding	Missense	SNV	c.418A>C	p.S140R	5026 (81%)	250 (5%)	2.5	6.2
**CHIP14**	CD34+ BM	PTPN11	12	PTPN11:chr12:112891084:A/C	Coding	Missense	SNV	c.418A>C	p.S140R	5856 (78%)	186 (3%)	1.6	6.2

**CHIP8**	CD34+ BM	PTPN11	12	PTPN11:chr12:112891084:A/C	Coding	Missense	SNV	c.418A>C	p.S140R	10982 (86%)	187 (2%)	0.9	4.3
**CHIP12**	CD34+ PB	PTPN11	12	PTPN11:chr12:112891084:A/C	Coding	Missense	SNV	c.418A>C	p.S140R	3593 (66%)	253 (7%)	3.5	7.7
**CHIP12**	CD34+ BM	PTPN11	12	PTPN11:chr12:112891084:A/C	Coding	Missense	SNV	c.418A>C	p.S140R	3451 (86%)	43 (1%)	0.6	5.1
**CHIP11**	CD34+ BM	PTPN11	12	PTPN11:chr12:112891084:A/C	Coding	Missense	SNV	c.418A>C	p.S140R	775 (75%)	24 (3%)	1.5	6.4
**CHIP9**	CD34+ PB	PTPN11	12	PTPN11:chr12:112891098:T/G	Coding	Synonymous	SNV	c.432T>G	p.P144=	2054 (74%)	125 (6%)	3.0	6.5
**CHIP1**	CD34- PB	PTPN11	12	PTPN11:chr12:112926935:C/T	NMD, coding(2)	Nonsense	SNV	c.1555C>T	p.Q519*	94 (96%)	2 (2%)	1.1	3.5
**CHIP1**	CD34- PB	RUNX1	21	RUNX1:chr21:36171713:G/A	Coding	Synonymous	SNV	c.852C>T	p.S284=	90 (92%)	1 (1%)	0.6	2.4
**CHIP4**	CD34+ PB	RUNX1	21	RUNX1:chr21:36206776:T/G	Coding	Missense	SNV	c.736A>C	p.T246P	558 (62%)	10 (2%)	0.9	8.5
**CHIP3**	CD34+ PB	RUNX1	21	RUNX1:chr21:36206776:T/G	Coding	Missense	SNV	c.736A>C	p.T246P	816 (56%)	23 (3%)	1.4	8.0
**CHIP3**	CD34+ PB	RUNX1	21	RUNX1:chr21:36206787:T/G	Coding	Missense	SNV	c.725A>C	p.H242P	956 (66%)	23 (2%)	1.2	6.3
**CHIP4**	CD34+ PB	RUNX1	21	RUNX1:chr21:36206790:T/G	Coding	Missense	SNV	c.722A>C	p.H242P	484 (54%)	110 (23%)	11.4	12.8
**CHIP3**	CD34+ PB	RUNX1	21	RUNX1:chr21:36206792:T/G	Coding	Synonymous	SNV	c.720A>C	p.P240=	788 (54%)	647 (82%)	41.1	27.8
**CHIP11**	CD34+ PB	SF3B1	2	SF3B1:chr2:198266834:T/C	Coding	Missense	SNV	c.2098A>G	p.K700E	9040 (95%)	175 (2%)	1.0	1.6
**CHIP11**	CD34+ BM	SF3B1	2	SF3B1:chr2:198266834:T/C	Coding	Missense	SNV	c.2098A>G	p.K700E	986 (96%)	24 (2%)	1.3	2.1
**CHIP1**	CD34- PB	SF3B1	2	SF3B1:chr2:198267531:A/G	Coding	Missense	SNV	c.1826T>C	p.M609T	96 (98%)	1 (1%)	0.5	3.1
**CHIP1**	CD34- PB	SF3B1	2	SF3B1:chr2:198267543:C/A	Coding, splicing	Missense	SNV	c.1814G>T	p.G605V	59 (60%)	4 (7%)	3.4	6.9
**CHIP1**	CD34+ PB	TET2	4	TET2:chr4:106155185:C/G	Coding	Missense	SNV	c.86C>G	p.P29R	2904 (80%)	2706 (93%)	47.9	41.2
**CHIP1**	CD138- BM	TET2	4	TET2:chr4:106155185:C/G	Coding	Missense	SNV	c.86C>G	p.P29R	6498 (76%)	6415 (99%)	49.4	38.4
**CHIP7**	CD34+ PB	TET2	4	TET2:chr4:106155199:C/T	Coding	Missense	SNV	c.100C>T	p.L34F	4471 (90%)	4312 (96%)	50.0	50.7
**CHIP8**	CD34+ BM	TET2	4	TET2:chr4:106155490:A/G	Coding	Missense	SNV	c.391A>G	p.R131G	8071 (63%)	137 (2%)	0.8	2.1
**CHIP1**	CD34- PB	TET2	4	TET2:chr4:106156138:G/A	Coding	Missense	SNV	c.1039G>A	p.A347T	98 (100%)	1 (1%)	0.5	4.6
**CHIP1**	CD34+ PB	TET2	4	TET2:chr4:106157870:A/G	Coding	Missense	SNV	c.2771A>G	p.H924R	3229 (89%)	3063 (95%)	49.2	50.1
**CHIP1**	CD138- BM	TET2	4	TET2:chr4:106157870:A/G	Coding	Missense	SNV	c.2771A>G	p.H924R	7072 (83%)	6900 (98%)	48.9	49.4
**CHIP1**	CD34- PB	TET2	4	TET2:chr4:106158346:C/T	NMD, coding(2)	Nonsense	SNV	c.3247C>T	p.Q1083*	61 (62%)	1 (2%)	1.6	3.7
**CHIP1**	CD34- PB	TET2	4	TET2:chr4:106158427:A/G	Coding	Missense	SNV	c.3328A>G	p.K1110E	74 (76%)	1 (1%)	1.4	3.0
**CHIP9**	CD34+ PB	TET2	4	TET2:chr4:106162497:G/T	Coding, splicing, splicing-ACMG(1)	Missense	SNV	c.3411G>T	p.E1137D	1780 (65%)	50 (3%)	1.4	5.0
**CHIP9**	CD34+ PB	TET2	4	TET2:chr4:106164829:T/G	Coding	Missense	SNV	c.3697T>G	p.W1233G	1428 (52%)	56 (4%)	2.0	3.4

**CHIP9**	CD34+ PB	TET2	4	TET2:chr4:106180822:T/G	Coding	Missense	SNV	c.3850T>G	p.S1284A	2124 (77%)	221 (10%)	5.2	9.0
**CHIP3**	CD34+ PB	TET2	4	TET2:chr4:106190805:T/G	Coding	Synonymous	SNV	c.4083T>G	p.G1361=	860 (59%)	15 (2%)	0.9	5.3
**CHIP11**	BM post ASCT	TET2	4	TET2:chr4:106190805:T/G	Coding	Synonymous	SNV	c.4083T>G	p.G1361=	3948 (65%)	436 (11%)	5.5	9.7
**CHIP1**	CD138- BM	TET2	4	TET2:chr4:106190805:T/G	Coding	Synonymous	SNV	c.4083T>G	p.G1361=	5421 (63%)	323 (6%)	3.0	5.8
**CHIP14**	CD34+ PB	TET2	4	TET2:chr4:106190805:T/G	Coding	Synonymous	SNV	c.4083T>G	p.G1361=	3425 (55%)	216 (6%)	3.2	7.0
**CHIP14**	CD34+ BM	TET2	4	TET2:chr4:106190805:T/G	Coding	Synonymous	SNV	c.4083T>G	p.G1361=	4372 (58%)	369 (8%)	4.2	8.6
**CHIP12**	CD34+ BM	TET2	4	TET2:chr4:106190805:T/G	Coding	Synonymous	SNV	c.4083T>G	p.G1361=	2788 (69%)	154 (6%)	2.8	5.7
**CHIP11**	CD34+ PB	TET2	4	TET2:chr4:106190805:T/G	Coding	Synonymous	SNV	c.4083T>G	p.G1361=	6362 (67%)	105 (2%)	0.8	2.7
**CHIP11**	CD34+ BM	TET2	4	TET2:chr4:106190805:T/G	Coding	Synonymous	SNV	c.4083T>G	p.G1361=	689 (67%)	73 (11%)	5.3	8.8
**CHIP9**	CD34+ PB	TET2	4	TET2:chr4:106190821:C/T	Coding	Missense	SNV	c.4099C>T	p.P1367S	2652 (96%)	57 (2%)	1.1	1.8
**CHIP9**	CD34+ PB	TET2	4	TET2:chr4:106190834:T/G	Coding	Missense	SNV	c.4112T>G	p.V1371G	2151 (78%)	138 (6%)	3.2	8.0
**CHIP1**	CD34- PB	TET2	4	TET2:chr4:106190834:T/G	Coding	Missense	SNV	c.4112T>G	p.V1371G	58 (59%)	6 (10%)	5.2	10.8
**CHIP8**	CD34+ PB	TET2	4	TET2:chr4:106196253:A/C	Coding	Missense	SNV	c.4586A>C	p.Q1529P	2164 (75%)	29 (1%)	0.7	6.8
**CHIP12**	CD34+ PB	TET2	4	TET2:chr4:106196253:A/C	Coding	Missense	SNV	c.4586A>C	p.Q1529P	3715 (68%)	67 (2%)	0.9	6.7
**CHIP4**	CD34+ PB	TET2	4	TET2:chr4:106196283:A/C	Coding	Missense	SNV	c.4616A>C	p.Q1529P	594 (66%)	10 (2%)	0.8	5.1
**CHIP14**	CD34+ BM	TET2	4	TET2:chr4:106196283:A/C	Coding	Missense	SNV	c.4616A>C	p.Q1529P	4033 (54%)	199 (5%)	2.5	8.2
**CHIP8**	CD34+ PB	TET2	4	TET2:chr4:106196283:A/C	Coding	Missense	SNV	c.4616A>C	p.Q1529P	1742 (60%)	116 (7%)	3.3	9.0
**CHIP12**	CD34+ PB	TET2	4	TET2:chr4:106196283:A/C	Coding	Missense	SNV	c.4616A>C	p.Q1529P	3306 (61%)	205 (6%)	3.1	8.7
**CHIP12**	CD34+ BM	TET2	4	TET2:chr4:106196283:A/C	Coding	Missense	SNV	c.4616A>C	p.Q1529P	2019 (50%)	62 (3%)	1.5	6.1
**CHIP11**	CD34+ PB	TET2	4	TET2:chr4:106196283:A/C	Coding	Missense	SNV	c.4616A>C	p.Q1529P	5092 (54%)	163 (3%)	1.6	6.6
**CHIP11**	CD34+ PB	TET2	4	TET2:chr4:106196283:A/C	Coding	Missense	SNV	c.4616A>C	p.Q1529P	627 (61%)	35 (6%)	2.8	8.1
**CHIP9**	CD34+ PB	TET2	4	TET2:chr4:106196792:T/C	Coding	Missense	SNV	c.5125T>C	p.C1709R	1810 (66%)	52 (3%)	1.4	5.1
**CHIP6**	CD34+ PB	TET2	4	TET2:chr4:106196792:T/C	Coding	Missense	SNV	c.5125T>C	p.C1709R	2437 (83%)	67 (3%)	1.4	2.6
**CHIP7**	CD34+ PB	TET2	4	TET2:chr4:106196792:T/C	Coding	Missense	SNV	c.5125T>C	p.C1709R	3838 (77%)	136 (4%)	1.8	3.0
**CHIP4**	CD34+ PB	TET2	4	TET2:chr4:106196792:T/C	Coding	Missense	SNV	c.5125T>C	p.C1709R	759 (84%)	16 (2%)	1.1	2.7
**CHIP2**	CD34+ PB	TET2	4	TET2:chr4:106196792:T/C	Coding	Missense	SNV	c.5125T>C	p.C1709R	7423 (61%)	158 (2%)	1.1	2.7
**CHIP11**	BM post ASCT	TET2	4	TET2:chr4:106196792:T/C	Coding	Missense	SNV	c.5125T>C	p.C1709R	4934 (81%)	94 (2%)	1.0	2.8
**CHIP1**	CD34+ PB	TET2	4	TET2:chr4:106196792:T/C	Coding	Missense	SNV	c.5125T>C	p.C1709R	2503 (69%)	126 (5%)	2.5	3.9

**CHIP1**	CD138- BM	TET2	4	TET2:chr4:106196792:T/C	Coding	Missense	SNV	c.5125T>C	p.C1709R	5562 (65%)	195 (4%)	1.8	3.3
**CHIP14**	CD34+ PB	TET2	4	TET2:chr4:106196792:T/C	Coding	Missense	SNV	c.5125T>C	p.C1709R	4542 (73%)	174 (4%)	1.9	2.8
**CHIP14**	CD34+ BM	TET2	4	TET2:chr4:106196792:T/C	Coding	Missense	SNV	c.5125T>C	p.C1709R	5117 (68%)	256 (5%)	2.5	3.4
**CHIP8**	CD34+ PB	TET2	4	TET2:chr4:106196792:T/C	Coding	Missense	SNV	c.5125T>C	p.C1709R	2431 (84%)	76 (3%)	1.6	3.0
**CHIP8**	CD34+ BM	TET2	4	TET2:chr4:106196792:T/C	Coding	Missense	SNV	c.5125T>C	p.C1709R	7166 (56%)	169 (2%)	1.2	3.5
**CHIP12**	CD34+ PB	TET2	4	TET2:chr4:106196792:T/C	Coding	Missense	SNV	c.5125T>C	p.C1709R	4032 (74%)	148 (4%)	1.8	3.2
**CHIP12**	CD34+ BM	TET2	4	TET2:chr4:106196792:T/C	Coding	Missense	SNV	c.5125T>C	p.C1709R	3276 (81%)	95 (3%)	1.4	2.8
**CHIP11**	CD34+ PB	TET2	4	TET2:chr4:106196792:T/C	Coding	Missense	SNV	c.5125T>C	p.C1709R	5944 (63%)	483 (8%)	4.1	4.4
**CHIP11**	CD34+ BM	TET2	4	TET2:chr4:106196792:T/C	Coding	Missense	SNV	c.5125T>C	p.C1709R	699 (68%)	79 (11%)	5.7	5.2
**CHIP6**	CD34+ PB	TET2	4	TET2:chr4:106196829:T/G	Coding	Missense	SNV	c.5162T>G	p.L1721W	2936 (100%)	2936 (100%)	99.9	99.7
**CHIP7**	CD34+ PB	TET2	4	TET2:chr4:106196829:T/G	Coding	Missense	SNV	c.5162T>G	p.L1721W	4667 (94%)	4479 (96%)	50.1	49.6
**CHIP4**	CD34+ PB	TET2	4	TET2:chr4:106196829:T/G	Coding	Missense	SNV	c.5162T>G	p.L1721W	875 (97%)	832 (95%)	49.7	50.8
**CHIP3**	CD34+ PB	TET2	4	TET2:chr4:106196829:T/G	Coding	Missense	SNV	c.5162T>G	p.L1721W	1406 (97%)	1345 (96%)	50.6	51.1
**CHIP2**	CD34+ PB	TET2	4	TET2:chr4:106196829:T/G	Coding	Missense	SNV	c.5162T>G	p.L1721W	11925 (98%)	11747 (99%)	50.0	49.9
**CHIP14**	CD34+ PB	TET2	4	TET2:chr4:106196829:T/G	Coding	Missense	SNV	c.5162T>G	p.L1721W	6091 (98%)	6091 (100%)	99.9	99.5
**CHIP14**	CD34+ BM	TET2	4	TET2:chr4:106196829:T/G	Coding	Missense	SNV	c.5162T>G	p.L1721W	7448 (99%)	7448 (100%)	100.0	99.5
**CHIP7**	CD34+ PB	TET2	4	TET2:chr4:106197000:A/G	Coding	Missense	SNV	c.5333A>G	p.H1778R	4688 (95%)	4495 (96%)	49.9	52.7
**CHIP1**	CD34+ PB	TET2	4	TET2:chr4:106197000:A/G	Coding	Missense	SNV	c.5333A>G	p.H1778R	3435 (95%)	3305 (96%)	50.0	52.9
**CHIP1**	CD138- BM	TET2	4	TET2:chr4:106197000:A/G	Coding	Missense	SNV	c.5333A>G	p.H1778R	8280 (97%)	8253 (100%)	50.1	51.1
**CHIP9**	CD34+ PB	TET2	4	TET2:chr4:106197002:G/A	Coding	Missense	SNV	c.5335G>A	p.A1779T	1418 (51%)	50 (4%)	1.8	6.1
**CHIP1**	CD34+ PB	TET2	4	TET2:chr4:106197139:T/G	Coding	Synonymous	SNV	c.5472T>G	p.G1824=	53 (54%)	11 (21%)	10.4	7.9
**CHIP14**	CD34+ BM	TET2	4	TET2:chr4:106197139:T/G	Coding	Synonymous	SNV	c.5472T>G	p.G1824=	4093 (54%)	77 (2%)	0.9	7.9
**CHIP1**	CD34- PB	TET2	4	TET2:chr4:106197172:T/G	Coding	Synonymous	SNV	c.5505T>G	p.G1835=	49 (50%)	19 (39%)	19.4	12.3
**CHIP8**	CD34+ PB	TET2	4	TET2:chr4:106197172:T/G	Coding	Synonymous	SNV	c.5505T>G	p.G1835=	1561 (54%)	30 (2%)	1.0	9.1
**CHIP9**	CD34+ PB	TET2	4	TET2:chr4:106197204:T/G	Coding	Missense	SNV	c.5537T>G	p.V1846G	1388 (50%)	42 (3%)	1.5	3.8
**CHIP9**	CD34+ PB	TP53	17	TP53:chr17:7574022:A/C	Coding	Synonymous	SNV	c.888T>G	p.R296=	1619 (59%)	75 (5%)	2.3	6.7

**CHIP9**	CD34+ PB	TP53	17	TP53:chr17:7574028:A/C	Coding, splicing	Synonymous	SNV	c.882T>G	p.R294=	2064 (75%)	73 (4%)	1.8	7.5
**CHIP9**	CD34+ PB	TP53	17	TP53:chr17:7576896:T/G	Coding	Missense	SNV	c.833A>C	p.Q278P	1403 (51%)	69 (5%)	2.5	5.4
**CHIP1**	CD138- BM	TP53	17	TP53:chr17:7577099:C/G	Coding	Missense	SNV	c.722G>C	p.R241T	7576 (89%)	243 (3%)	1.6	2.2
**CHIP1**	CD138- BM	TP53	17	TP53:chr17:7577127:C/T	Coding	Missense	SNV	c.694G>A	p.E232K	7819 (92%)	109 (1%)	0.7	2.0
**CHIP9**	CD34+ PB	TP53	17	TP53:chr17:7577500:T/G	Coding, splicing, splicing-ACMG[(1)]	Missense	SNV	c.664A>C	p.S222R	2151 (78%)	39 (2%)	0.9	5.4
**CHIP8**	CD34+ PB	TP53	17	TP53:chr17:7578210:T/C	Coding	Synonymous	SNV	c.522A>G	p.R174=	2442 (85%)	2293 (94%)	50.2	51.6
**CHIP9**	CD34+ PB	TP53	17	TP53:chr17:7578434:A/C	Coding	Missense	SNV	c.379T>G	p.S127A	1441 (52%)	33 (2%)	1.1	4.3
**CHIP1**	CD34- PB	TP53	17	TP53:chr17:7578460:A/G	Coding	Missense	SNV	c.353T>C	p.V118A	54 (55%)	1 (2%)	0.9	1.6
**CHIP8**	CD34+ PB	TP53	17	TP53:chr17:7578480:T/G	Coding	Synonymous	SNV	c.333A>C	p.T111=	1639 (57%)	38 (2%)	1.2	7.5
**CHIP1**	CD138- BM	TP53	17	TP53:chr17:7578508:C/T	Coding	Missense	SNV	c.305G>A	p.C102Y	6029 (71%)	91 (2%)	0.8	2.3
**CHIP1**	CD34+ PB	U2AF1	21	U2AF1:chr21:44514601:C/T	Coding	Synonymous	SNV	c.555G>A	p.L185=	3246 (90%)	3045 (94%)	49.6	50.5
**CHIP1**	CD138- BM	U2AF1	21	U2AF1:chr21:44514601:C/T	Coding	Synonymous	SNV	c.555G>A	p.L185=	7550 (88%)	7480 (99%)	49.9	50.3
**CHIP1**	CD34- PB	U2AF1	21	U2AF1:chr21:44514601:C/T	Coding	Synonymous	SNV	c.555G>A	p.L185=	86 (88%)	78 (91%)	45.9	45.2
**CHIP1**	CD34- PB	WT1	11	WT1:chr11:32413520:G/A	Coding, splicing	Missense	SNV	c.1445C>T	p.T482I	97 (99%)	1 (1%)	0.5	4.0
**CHIP1**	CD34- PB	WT1	11	WT1:chr11:32413605:TCA/T	NMD, coding, splicing[(2)]	Frameshift	Deletion	c.1358_1359del	p.V453Efs*6	97 (99%)	2 (2%)	1.0	2.0
**CHIP9**	CD34+ PB	WT1	11	WT1:chr11:32417899:T/G	Coding	Missense	SNV	c.1168A>C	p.T390P	1506 (55%)	85 (6%)	2.8	5.9
**CHIP7**	CD34+ PB	WT1	11	WT1:chr11:32417945:T/C	Coding, splicing	Synonymous	SNV	c.1122A>G	p.R374=	4583 (92%)	4496 (98%)	50.1	50.6
**CHIP1**	CD34+ PB	WT1	11	WT1:chr11:32417945:T/C	Coding, splicing	Synonymous	SNV	c.1122A>G	p.R374=	3365 (93%)	3300 (98%)	50.1	50.4
**CHIP1**	CD138- BM	WT1	11	WT1:chr11:32417945:T/C	Coding, splicing	Synonymous	SNV	c.1122A>G	p.R374=	8074 (94%)	8062 (100%)	50.0	49.7
**CHIP1**	CD34- PB	WT1	11	WT1:chr11:32417945:T/C	Coding, splicing	Synonymous	SNV	c.1122A>G	p.R374=	79 (81%)	76 (96%)	48.7	40.8
**CHIP14**	CD34+ PB	WT1	11	WT1:chr11:32417945:T/C	Coding, splicing	Synonymous	SNV	c.1122A>G	p.R374=	5225 (84%)	5225 (100%)	100.0	99.9
**CHIP14**	CD34+ BM	WT1	11	WT1:chr11:32417945:T/C	Coding, splicing	Synonymous	SNV	c.1122A>G	p.R374=	7198 (96%)	7198 (100%)	99.9	99.9
**CHIP8**	CD34+ PB	WT1	11	WT1:chr11:32417945:T/C	Coding, splicing	Synonymous	SNV	c.1122A>G	p.R374=	2630 (91%)	2534 (96%)	49.9	50.7
**CHIP8**	CD34+ BM	WT1	11	WT1:chr11:32417945:T/C	Coding, splicing	Synonymous	SNV	c.1122A>G	p.R374=	9382 (74%)	9329 (99%)	50.0	49.2

[(1)] According to *the American College of Medical Genetics and Genomics-Association for Molecular Pathology* (ACMG-AMP) interpretation guidelines

[(2)] Nonsense-mediated decay (NMD)

Supplementary Table S3. Called pathogenic somatic Variants. All pathogenic somatic coding variants identified in all samples analyzed (n=19). SNV=Single Nucleotide Variant.

Sample	Sample Type	Gene	Chromosome	Variant	Function	Coding Impact	Variant Type	cDNA	Protein Change	Genotyped Cells	Mutated Cells	VAF by cell count (%)	VAF by read count (%)
**CHIP11**	BM post ASCT	DNMT3A	2	DNMT3A:chr2:25457242:C/T	Coding	Missense	SNV	c.2645G>A	p.R882H	5376 (88%)	3985 (74%)	39.6	38.5
**CHIP11**	CD34+ PB	DNMT3A	2	DNMT3A:chr2:25457242:C/T	Coding	Missense	SNV	c.2645G>A	p.R882H	6843 (72%)	5312 (78%)	41.3	37.1
**CHIP11**	CD34+ BM	DNMT3A	2	DNMT3A:chr2:25457242:C/T	Coding	Missense	SNV	c.2645G>A	p.R882H	837 (81%)	521 (62%)	35.2	34.5
**CHIP9**	CD34+ PB	DNMT3A	2	DNMT3A:chr2:25457246:T/G	Coding	Missense	SNV	c.2641A>C	p.S881R	1661 (60%)	320 (19%)	9.6	13.4
**CHIP1**	CD34- PB	DNMT3A	2	DNMT3A:chr2:25462086:T/G	Intronic, splicing, splicing-ACMG[(1)]		SNV	c.2323-2A>C		61 (62%)	5 (8%)	4.1	4.6
**CHIP7**	CD34+ PB	DNMT3A	2	DNMT3A:chr2:25463182:G/A	NMD, coding[(2)]	Nonsense	SNV	c.2311C>T	p.R771*	4758 (96%)	134 (3%)	1.5	1.9
**CHIP14**	CD34+ PB	DNMT3A	2	DNMT3A:chr2:25463248:G/A	Coding	Missense	SNV	c.2245C>T	p.R749C	4973 (80%)	121 (2%)	1.2	3.3
**CHIP14**	CD34+ BM	DNMT3A	2	DNMT3A:chr2:25463248:G/A	Coding	Missense	SNV	c.2245C>T	p.R749C	6361 (85%)	100 (2%)	0.8	3.0
**CHIP1**	CD138- BM	EZH2	7	EZH2:chr7:148512080:T/G	Coding	Missense	SNV	c.1598A>C	p.Q533P	5084 (60%)	182 (4%)	1.8	4.8
**CHIP1**	CD34+ PB	GATA2	3	GATA2:chr3:128200690:G/A	Coding	Missense	SNV	c.1115C>T	p.A372V	2549 (70%)	51 (2%)	2.4	1.6
**CHIP1**	CD138- BM	GATA2	3	GATA2:chr3:128200690:G/A	Coding	Missense	SNV	c.1115C>T	p.A372V	5010 (59%)	127 (3%)	1.3	2.6
**CHIP9**	CD34+ PB	KIT	4	KIT:chr4:55599336:C/A	Coding	Missense	SNV	c.2462C>A	p.S821Y	1463 (53%)	926 (63%)	31.6	23.8
**CHIP12**	CD34+ PB	KIT	4	KIT:chr4:55599336:C/A	Coding	Missense	SNV	c.2462C>A	p.S821Y	3424 (63%)	533 (16%)	7.8	14.4
**CHIP12**	CD34+ BM	KIT	4	KIT:chr4:55599336:C/A	Coding	Missense	SNV	c.2462C>A	p.S821Y	2500 (62%)	185 (7%)	3.7	7.4
**CHIP11**	CD34+ BM	NRAS	1	NRAS:chr1:115256529:T/C	Coding	Missense	SNV	c.182A>G	p.Q61R	945 (92%)	19 (2%)	1.1	2.3
**CHIP11**	CD34+ PB	SF3B1	2	SF3B1:chr2:198266834:T/C	Coding	Missense	SNV	c.2098A>G	p.K700E	9040 (95%)	175 (2%)	1.0	1.6
**CHIP11**	CD34+ BM	SF3B1	2	SF3B1:chr2:198266834:T/C	Coding	Missense	SNV	c.2098A>G	p.K700E	986 (96%)	24 (2%)	1.3	2.1
**CHIP14**	CD34+ BM	TET2	4	TET2:chr4:106196283:A/C	Coding	Missense	SNV	c.4616A>C	p.Q1529P	4033 (54%)	199 (5%)	2.5	8.2

(1) According to *the American College of Medical Genetics and Genomics-Association for Molecular Pathology* (ACMG-AMP) interpretation guidelines

(2) Nonsense-mediated decay (NMD)

Supplementary Table S4. Patients cytogenic characteristics.

	Total (n=12)	Presence of CHIP mutation (n=6)	Absence of CHIP mutation (n=6)
t(4;14)			
Yes	2 (17%)	1 (17%)	1 (17%)
No	8 (66%)	4 (66%)	4 (66%)
Not available	2 (17%)	1 (17%)	1 (17%)
t(11;14)			
Yes	3 (25%)	3 (50%)	0
No	4 (33%)	2 (33%)	2 (33%)
Not available	5 (42%)	1 (17%)	4 (67%)
t(14;16)			
Yes	0	0	0
No	10 (83%)	5 (83%)	5 (83%)
Not available	2 (17%)	1 (17%)	1 (17%)
t(14;20)			
Yes	0	0	0
No	7 (58%)	5 (83%)	2 (33%)
Not available	5 (42%)	1 (17%)	4 (67%)
Del17p			
Yes	0	0	0
No	10 (83%)	5 (83%)	5 (83%)
Not available	2 (17%)	1 (17%)	1 (17%)
Amp1q			
Yes	7 (58%)	3 (50%)	4 (66%)
No	3 (25%)	2 (33%)	1 (17%)
Not available	2 (17%)	1 (17%)	1 (17%)
De1p			
Yes	0	0	0
No	9 (75%)	5 (83%)	4 (67%)
Not available	3 (25%)	1 (17%)	2 (33%)

Patients' cytogenetic characteristics were assessed using the standard technique of Fluorescence in situ Hybridization (FISH).
